# Web conferencing systems: Skype and MSN in telepathology

**DOI:** 10.1186/1746-1596-3-S1-S13

**Published:** 2008-07-15

**Authors:** Clóvis Klock, Regina de Paula Xavier Gomes

**Affiliations:** 1 Hospital Santa Teresinha, Erechim – RS, Brazil; 2 Laboratório CPD – Citologia e Patologia Diagnosticas Ltda, Curitiba, PR, Brazil

## Abstract

Virtual pathology is a very important tool that can be used in several ways, including interconsultations with specialists in many areas and for frozen sections. We considered in this work the use of Windows Live Messenger and Skype for image transmission. The conference was made through wide broad internet using Nikon E 200 microscope and Digital Samsung Colour SCC-131 camera. Internet speed for transmission varied from 400 Kb to 2.0 Mb. Both programs allow voice transmission concomitant to image, so the communication between the involved pathologists was possible using microphones and speakers. Alive image could be seen by the receptor pathologist who was able to ask for moving the field or increase/diminish the augmentation. No phone call or typing required. The programs MSN and Skype can be used in many ways and with different operational systems installed in the computer. The capture system is simple and relatively cheap, what proves the viability of the system to be used in developing countries and in cities where do not exist pathologists. With the improvement of software and the improvement of digital image quality, associated to the use of the high speed broad band Internet this will be able to become a new modality in surgical pathology.

## Background

Virtual pathology is a very important tool that can be used in several ways. Amongst them we have interconsultations with specialists in many areas and the exchange of opinions between colleagues. The use in cases of frozen sections is still cited where one trained technician can carry through the macroscopic procedure and the preparation of slides and the pathologist in another place will be able to give the diagnosis [1]. We consider, in this work, the use of two available tools, the MSN and the Skype.

## Methods

**Figure 1:**
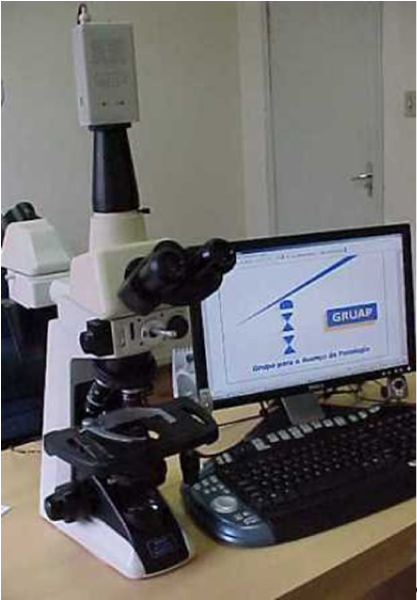
Our telepathology system.

T capture the images we used a microscope Nikon E 400, trinocular and double head Y-THF, with a Digital Samsung Colour SCC-131 camera. The camera image was transmitted to a personal computer Quad-Core Intel^® ^3.4 GHz Xeon processor, Hard disk SATA of 1 Tb, 7.200 RPM, and Windows XP SP2 as the operating system. The capture plaque was Hauppauge NTSC M with its supporting software (Hauppauge WinTV Capture) (fig. 1). The computer was connected to the internet by ADSL system, with speed varying from 400 Kb to 2.0 Mb. The pathologists on both sides were designated transmitter (T) and receptor (R).

### Windows Live Messenger

**Figure 2:**
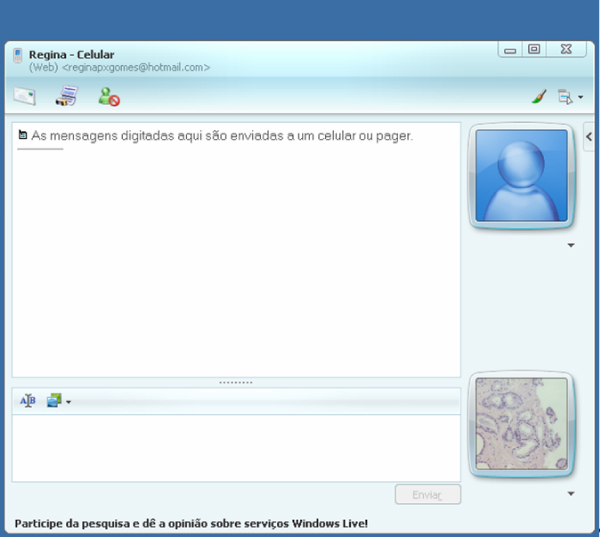
MSN System.

The software Windows Live Messenger (version 8.1, compilation 8.1.0178.00), Microsoft Network^©^, commonly designated MSN, was installed and accessed through personal identification with e-mail address and password (fig. 2).

### Skype™

**Figure 3:**
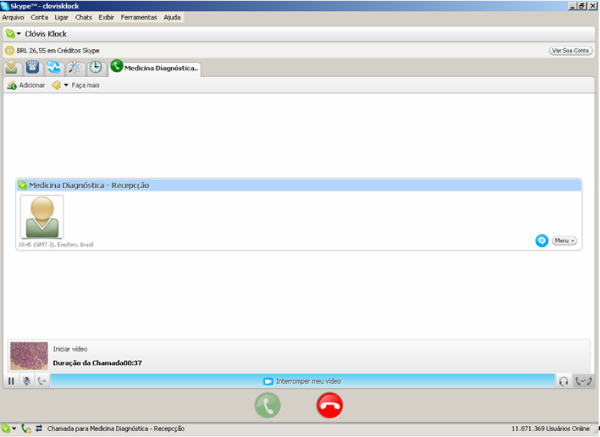
Skype System.

The Software Skype™, Skype Limited^©^, version 3.5.0.229, Joltid™ Limited, was alsp accessed through personal identification and password (fig. 3).

Both Skype and MSN allow voice transmission concomitant to image. So, for communication between T and R pathologists, microphones and speakers were necessary.

## Results

After establishment of the connection between the two pathologists, a live image could be seen by the receptor pathologist who was able to ask for moving the field or to increase/diminish the augmentation. Usually the transmitter showed a low power field image and then increased the magnification as needed. All the dialogues were through the MSN and Skype system, with microphones, with no phone call or typing required.

## Discussion

The software Skype was created in 2003 by two entrepreneurs from Sweden and Denmark, Niklas Zennström and Janus Friis, and by a team of software developers, Ahti Heinla, Priit Kasesalu and Jaan Tallinn based in Tallinn, Estonia [2]. It allows users to make phone calls over the Internet to other computers with the Skype software, or even to conventional telephones and cell phones, these for a fee that is usually lower than the ones normally used. The software also allows the user to transfer files and send instant written messages. Videoconferencing was introduced in 2006 for the Windows and Mac OS X platform clients and in 2007 for Linux version 2.0. The first version used for videoconferencing, 3.6.0.216, supports "High Quality Video" with quality and features (full-screen and screen-in-screen modes) claimed to be similar to middle-range video-conferencing systems. Versions for Microsoft Windows 2000, XP, Vista and Windows Mobile, Mac OS X (Intel and PPC) and Linux (32-bit ×86 only) are now available. Skype can be run from a USB stick without being installed on the target computer in the Windows system. Skype is reportedly willing to accept thousands of connections, but is stated to limit itself to 40 Kb/s upload and download. The communication is considered secure and encryption cannot be disabled without the user seeing. One has to remember that Skype provides an uncontrolled registration system for users with absolutely no proof of identity, so the displayed caller's name is no guarantee of authenticity [3].

The concept for messenger (Windows Live Messenger, MSN) was created by the Advanced Technology Group at Microsoft, headed by Nathan Myhrvold. MSN was originally a dial-up online content provider. Service was initially included with Windows 95 installations for simple written conversation as a chat. Nowadays, the new version allows the user to transfer files, share many folders with a specified user, have on line conversations, play games, and remotely access another computer [4].

The programs MSN and Skype can be used for discussion of cases, second opinion, or even, in the case of Skype, for a video conference (chat) with the participation of some specialists in the most varied localization. In both systems you can create a conference with many users. These functions are very important when you need to participate in a meeting and you can't be physically in the place, or if you need, as in the case of pathologists, a second opinion on a case and you don't have enough time. A good example is frozen sections that some authors have described being done through this method. One person does the frozen sections, slides and the transmission and the pathologist, in any place, can see the images and give a diagnosis.

## Conclusion

The capture system with MSN and Skype is simple, which proves the viability of the system to be used in developing countries and in places where there are no pathologists. Some colleagues have already been able to test this new technique of information exchange and visualization of images in real time. With the improvement of software and the improvement of digital image quality, associated with the use of the high speed broad band Internet (above of 2.0 Mb), this will be able to become a new modality in surgical pathology.
